# Bibliographical

**Published:** 1873-06

**Authors:** 


### BIBLIOGRAPHICAL.

Text-Book of Physiology, General, Special and Practical. By John Hughes Bennett, M.D., F.R.C.S., Professor of the Institutes of Medicine or Physiology, and Senior Professor of Clinical Medicine in the University of Edinburgh, etc., etc. With Twenty-five Photo-lithographic Plates. Philadelphia: J. B. Lippincott & Co. 1873.

This work will be found a valuable accession to the libraries of students and practitioners, for the reason that in the shortest time possible they may reach the pith of any question in physiology. The author says: "I have hitherto hesitated to write a text-book, apprehensive that the extensive field, theoretical and practical, now occupied by the science, would render the work too bulky. In yielding, therefore, at length to the earnest and repeated requests of my classes to furnish them with a book that would aid their studies, it has to be seen how far the attempt to condense in this volume so comprehensive a science will satisfy the expectations and meet the requirements of the physiological student."

It is a good book, well up to the times, so condensed and practically arranged as to meet the objects of its publication in supplying a want long felt by the student and busy practitioner.

Diseases of the Ovaries, their Diagnosis and Treatment. By T. Spencer Wells, Fellow and Member of the Royal College of Surgeons of England, etc., etc., etc. New York: D. Appleton & Co., 549 and 551 Broadway. 1873.

Among authors upon diseases of the ovaries, T. Spencer Wells occupies no second place, and the volume now issued by the Appletons will serve to give American readers a more intimate acquaintance with the views of one whose devotion to a specialty of vast importance to the profession has accomplished so much for the exaltation of science and the welfare of woman. Within limits so necessarily restricted as our notice must be, we can but call attention to the value of the work by referring to its table of contents: Chapter I. Anatomical and physiological notes on the pelvic organs. Chapter II. Morbid anatomy and pathology of the ovaries. Chapter III. Dermoid cysts. Chapter IV. Inflammation, degeneration and rotation of ovarian tumors; physiognomy of patients with ovarian disease. Chapter V. Contents of ovarian cysts. Chapter VI. Diagnosis of ovarian tumors. Chapter VII. On the mode of investigating and recording cases. Chapter VIII. The medical treatment of ovarian tumors. Chapter IX. On the treatment of ovarian cysts by abdominal tapping, vaginal tapping, vaginal tapping and drainage, injection of iodine and incision. Chapter X. The rise and progress of ovariotomy. Chapter XI. On the selection of cases for ovariotomy. Chapter YII. Preparation of a patient for ovariotomy; duties of the nurse; description of the necessary instruments. Chapter XIII. The operation of ovariotomy; division of the abdominal wall; situation and length of incision. Chapter XIV. Erupting, separation and removal of the cyst or tumor. Chapter XV. Treatment of the pedicle, sponging of the peritoneum and closure of the wound. Chapter XVI. On the treatment of patient after ovariotomy. Chapter XVII. On the removal of both ovaries at one operation. Chapter XVIII. On ovariotomy performed twice on the same subject. Chapter XIX. On incomplete ovariotomy aud exploratory incisions. Chapter XX. Results of ovariotomy, and subsequent history of patients who recovered.

Manual of Chemical Analysis as applied to the examination of Medicinal Chemicals. A Guide for the Determination of their Identity and Quality, and for the Detection of Impurities and Adulterations. For the use of Pharmaceutists, Physicians, Druggists and Manufacturing Chemists, and of Pharmaceutical Medical Students. By Fredrick Hoffmann, Ph. D. Pharmaceutist in New York. New York: D. Appleton & Co. 1873.

The value of this work is readily seen. So far as the physician is concerned, he has two means at his command of guarding his patients against the numerous adultertions of drugs. The first is to purchase good articles, without reference to price, from responsible druggists. Cheap drugs are humbugs. The second is to test the purity of the drugs purchased by the practical application of the methods employed in the manual under notice. For this purpose it is invaluable, and will be found complete and satisfactory.

The Pharmacopœia of the United States. Fifth Decennial Revision. By Author-of the National Canvention for Revising the Pharmacopœia, held at Washington, A.D. 1870. Philadelphia: J. B. Lippincott & Co. 1873.

The Pharmacopœia is indispensable to the druggist, and the country physician who engages in office pharmacy to any extent will be greatly benefited by having a copy for guidance and instruction.

Mineral Springs of the United States and Canada: with Analysis and Notes on the Prominent Spas of Europe and a list of Sea-side Resorts. By Geo. E. Walton, M.D., Lecturer on Materia Medica in the Miami Medical College, Cincinnati, etc., etc. New York: D. Appleton & Co. 1873.

This volume contains much useful information to be found no where else. Many of the famous mineral springs are noticed, and while the list is not complete, it is a creditable effort in the right direction. Only three springs are noticed in Georgia, notwithstanding the virtues of her mineral waters are unsurpassed by springs of other sections. A few of our springs deserve more than a passing notice at our hands, had we the time or space to devote to it. Harrison's Springs, near Dahlonega, and those in the vicinity of Atlanta, are worthy of special note. Ponce de Leon Spring should, by all means, occupy a prominent place in the next edition of Dr. Walton's work.

Transactions of the Third Annual Session of the Medical Society of Virginia. 1872.

The volume before us is one of very great interest and value. The annual address of the President, Dr. A. M. Fauntleroy, "On the Vis Medicatrix Nature," and the address of Dr. Landon B. Edwards, "On Medical Ignorance and Medical Reforms," are worthy of careful reading. The lengthy paper of our esteemed associate, Dr. A. S. Payne, "On Epidemics of Piedmont District for the years 1840 and 1872 inclusive, with remarks upon the Topography, Hydrography, Petrology, etc., of the District," is one of the best of the volume, and one our readers would do well to peruse. The "Report of the Committee on Epidemics of Tidewater District" is a paper of great merit. The volume contains papers by Dr. Thos. P. Atkinson, on "Differences between the

White and the Black Races;" by Dr. Wm. B. Gray, "On the Hypodermic use of Sulphate Strychnia as an Optic Nerve Stimulant;" by Dr. John E. Apperson, on "Large Pelvic Abscess;" by Dr. Wm. D. Hooper, on "A Case of Extra-uterine Pregnancy—Removal of the Fœtal Skeleton by Abdominal Incision—Recovery;" by Dr. O. B. Jenks, on "A Novel Case of Extreme Mobility of the Knee-joint in a New-born Child;" by Dr. F. D. Cunningham, on "Defective Vision and the Principles on which it may be Corrected by Optic Means;" by Drs. W. W. Parker, D. W. Lassiter, and R. S. Hamilton, Committee, on "The Effect of the use of the Sewing Machine on the Health of Women;" by Dr. William D. Hooper, on "New Method of Treating Compound Fractures and Stumps after Amputation, with eight illustrated cases;" by Dr. J. Herbert Claiborne, on "Diphtheria, Cases and Notes"—all of which make up a volume of rare excellence—one we hope our friends every where may secure.

Clinical Lectures on Various Important Diseases: being a Collection of the Clinical Lectures delivered in the Medical Wards of Mercy Hospital, Chicago, by Nathan S. Davis, A.M., M.D., Professor of Principles and Practice of Medicine and Clinical Medicine in Chicago Medical College. Edited by Frank H. Davis, M.D. Chicago: J. J. Spalding & Co., 158 Clark Street. 1873.

We have often enriched our pages with portions of the clinical lectures of Professor Davis. We assure our readers we have seldom met with lectures containing more true medical philosophy, simply but delightfully told, than in the little volume before us. Any one of the eighteen lectures is worth the price of the book, and we desire to express the hope that it will be found on the shelves of every reader of the Record.

